# Recent Discoveries in Epigenetic Modifications of Polycystic Kidney Disease

**DOI:** 10.3390/ijms222413327

**Published:** 2021-12-11

**Authors:** Sarah A. Bowden, Euan J. Rodger, Aniruddha Chatterjee, Michael R. Eccles, Cherie Stayner

**Affiliations:** 1 Department of Pathology, Dunedin School of Medicine, University of Otago, 270 Great King Street, Dunedin 9054, New Zealand; thisissarahbowden@gmail.com (S.A.B.); euan.rodger@otago.ac.nz (E.J.R.); aniruddha.chatterjee@otago.ac.nz (A.C.); michael.eccles@otago.ac.nz (M.R.E.); 2 Maurice Wilkins Centre for Molecular Biodiscovery, Level 2, 3A Symonds Street, Auckland 1010, New Zealand

**Keywords:** epigenetics, epigenome, polycystic kidney disease, DNA methylation, cystogenesis, inherited kidney disease, ADPKD, *PKD1*

## Abstract

Autosomal Dominant Polycystic Kidney Disease (ADPKD) is a heritable renal disease that results in end-stage kidney disease, due to the uncontrolled bilateral growth of cysts throughout the kidneys. While it is known that a mutation within a PKD-causing gene is required for the development of ADPKD, the underlying mechanism(s) causing cystogenesis and progression of the disease are not well understood. Limited therapeutic options are currently available to slow the rate of cystic growth. Epigenetic modifications, including DNA methylation, are known to be altered in neoplasia, and several FDA-approved therapeutics target these disease-specific changes. As there are many similarities between ADPKD and neoplasia, we (and others) have postulated that ADPKD kidneys contain alterations to their epigenetic landscape that could be exploited for future therapeutic discovery. Here we summarise the current understanding of epigenetic changes that are associated with ADPKD, with a particular focus on the burgeoning field of ADPKD-specific alterations in DNA methylation.

## 1. Introduction

Autosomal Dominant Polycystic Kidney Disease (ADPKD) is the most common heritable renal disease in humans. Characterised by the development of large, fluid-filled cysts in both kidneys, this disease has an estimated prevalence of three cases per 10,000 [1]. As cysts continue to develop and grow throughout a patient’s lifetime, the kidney will ultimately enlarge from less than 200 g in a healthy person to upwards of 1.5 kg in some ADPKD patients. The amount of cystic growth (both by number and volume) varies between patients, from those who develop only a few cysts and have adequate renal function into late adulthood [2], to patients with cysts visible as early as in utero, and who experience severe and rapidly progressive disease [3].

As a consequence of cyst growth, renal function is impaired. It is assumed that either the destruction of the renal parenchyma [4], the fibrosis of interstitial kidney tissue [5], the disruption of the renal architecture [6,7], or a combination of any of these precludes the normal functioning of nephrons [8]. Patients present with symptoms that reflect the decline of renal function, including flank pain, hypertension and various urinary complications. Consistent with the widespread expression of the causative genes, ADPKD patients often have extra-renal abnormalities and can experience several cardiac-related conditions. Patients may develop hypertension up to ten years earlier than the average person (even when they exhibit adequate renal function). Common extra-renal symptoms include cysts in other organs such as the liver, pancreas, and intestines. The prevalence of intracranial aneurysms is also higher in ADPKD patients than in the general population [9,10]. Many of these extra-renal symptoms are caused by connective tissue defects [11].

ADPKD is associated with significant morbidity, and patients have a reduced life expectancy [12,13] when compared with that of the general population. Progression of ADPKD will ultimately inhibit renal function to the point of end-stage kidney disease (ESKD). An estimated 50% of ADPKD patients will reach ESKD by age 60 [12,14], at which point they typically require dialysis or renal transplantation. Despite the significant medical burden associated with ADPKD, there is currently no cure for this disease, and patients are treated with a limited number of therapeutics that elicit moderate improvement in some individuals. One of the recently available therapeutic options is the vasopressin V2-receptor antagonist tolvaptan, which slows fluid accumulation in renal cysts. This drug has now been approved for the treatment of ADPKD in several countries, as it attenuates the rate of cyst growth by half, delaying the onset of ESKD. However, undesirable side effects, such as abnormal liver function, has resulted in poor patient tolerance of tolvaptan [15]. There is a clear need for better treatments for the approximately 2.4 million people worldwide who are affected by ADPKD.

### 1.1. PKD-Causing Genes

ADPKD is genetically heterogeneous—a germline mutation within one of a handful of PKD-causing genes is a prerequisite for patients to develop the disease. *PKD1* is the most prevalent disease-causing gene for ADPKD, with approximately 85% of patients having mutations within this locus. Mutations in *PKD2* account for most of the remaining 15% of patients [16]. More recent whole-genome sequencing of patients with atypical ADPKD has led to the discovery of patients with mutations in novel genes including *GANAB* [17] and *DNAJB11* [18], which are now confirmed to cause mild forms of PKD in a small proportion of patients. Additionally, access to next-generation sequencing has revealed a suspected role for additional genes in patient pathology. Approximately 10% of patients have no family history of ADPKD, and only 25% of these have been shown to have *de novo* mutations in *PKD1* [19].

There is no mutation ‘hot-spot’ within *PKD1* or *PKD2;* mutations are highly variable, are spread across each gene, and are typically unique within families. A database of disease-causing mutations within *PKD1* and *PKD2* has been established (pkdb.mayo.edu (accessed on 30 October 2021)) [20]; over 2000 mutations have been identified as contributing to the pathogenicity in ADPKD, with frameshift mutations accounting for the largest percentage of predicted pathogenic mutations recorded to-date. The pattern of inheritance follows that of autosomal dominance, as most patients are heterozygotes for the pathogenic mutation they carry.

While these germline mutations correlate with the development of ADPKD, they are still insufficient to fully explain the process of cystogenesis. Although each kidney’s approximately one million nephrons all carry the germline PKD-causing mutation, less than 1% of these will develop cysts [21]. Various theories have been postulated to explain this process, the most common of which is loss of heterozygosity (LOH) [22,23,24,25]. Loss of heterozygosity (also called the two-hit hypothesis) was first identified as a mechanism for the accumulation of tumour-causing mutations in cancer [26]. Extensive data suggest that this mechanism also occurs in cyst development [25]. The presence of accumulating somatic *PKD1* or *PKD2* mutations can explain the initiation of cyst growth over time, and several studies have identified somatic “second hit” mutations in isolated cysts [24,27,28,29]. But not all cysts contain a somatic mutation in the second allele of the causative gene, and the evidence to date suggests a number of different mechanisms are at play, including gene dosage effects [30]. The net result (however it is achieved) is changes in gene expression, affecting many different signalling pathways [31].

### 1.2. ADPKD as ‘Neoplasia in Disguise’

The uncontrolled growth of cysts that occurs in ADPKD has clear parallels with cancer. Cysts are effectively benign neoplasms [12], and the disease itself has been described as *‘neoplasia in disguise’* [32]. Features of ADPKD align with eight of the ten hallmarks of cancer [33,34], and these similarities suggest a possibility of repurposing therapeutics currently developed for use in cancers for ADPKD, some of which have already been the subject of clinical trials in ADPKD [35].

An underexplored parallel between these two disease states includes changes in epigenetic modifications, which can result in large scale gene expression changes. Exploring epigenetic alterations in ADPKD may not only shed light on the process of cystogenesis and cyst growth, but could lead to new therapeutic options being available to slow the course of this disease.

## 2. Epigenetic and Post-Translational Modification in ADPKD

### 2.1. Potential Epigenetic Changes Associated with ADPKD

In addition to germline or somatic mutations, relatively stable alterations in gene expression can occur through epigenetic mechanisms. These are molecular changes that occur to the DNA molecule without changing the genetic sequence. While epigenetic mechanisms are essential for normal cellular function/development [36], these processes can be aberrantly altered, leading to (or as a result of) disease, and eliciting changes in gene expression.

During development, epigenetic mechanisms play a role in cellular growth and differentiation, and as cells mature, these epigenetic modifications change to suit the role of the cell. Once the cell differentiates, epigenetic modifications are specific for each cell type. These modifications (and any subsequent disease-causing epigenetic changes) can be inherited through progressive cell lineages.

Two key forms of epigenetic regulation are histone modification (such as acetylation and methylation of histone proteins) and DNA methylation. These mechanisms act to modify the chromatin state and accessibility of the DNA to transcription factors [36]. An associated biological mechanism that can also alter gene expression is the post-translational regulation of genes by microRNA. Evidence for alterations in the epigenetic control of gene expression in ADPKD is accumulating [37,38,39] and emerging data, particularly in the area of DNA methylation are explored below.

### 2.2. MicroRNA

MicroRNAs (miRNAs) are a class of non-coding RNAs that are approximately 20 nucleotides long. These sequences function as post-translational regulators to alter protein expression by destabilising mRNA translation [40]. It has been estimated that one third of protein coding genes may be regulated by miRNA [41], and in cancer it has been documented that a large number of genes are modified by specific miRNAs. Some notable cancer-associated genes that are regulated by miRNA include the anti-apoptosis gene *BCL2* and the oncogene *MYC* [42].

Previous research in renal development has confirmed that miRNAs are required for embryonic development of the kidney, and several miRNAs have been implicated in glomerular and tubular diseases [43]. Screening of miRNA in tissue and plasma samples has identified a wide range of miRNA as being dysregulated in ADPKD. These include the upregulation of miR-182-5p [44] and 199a-5p [45], as well as the downregulation of miR-192 and miR-194 (due to hypermethylation at these two loci) [46]. It has also been demonstrated that miR-501-5p is upregulated in ADPKD, causing activation of mTOR pathways through p53-mediated mechanisms [47].

Inhibition of the miR-17~92 cluster has been demonstrated to reduce cyst proliferation in ADPKD mice [48]. Further analysis of the six miRNAs within this cluster (miR-17, miR-18a, miR-19a, miR-20a, miR-19b-1, miR-92a-1) provide evidence that modulation of miR-17 alone is sufficient to attenuate the growth of cysts [49]. Additionally, the anti-miR-17 treatment in this study inhibited mTOR signalling. As mTOR has previously been assessed as a therapeutic target in ADPKD, inhibition of this miRNA suggests an additional treatment option, especially as clinical trials of the mTOR inhibitor rapamycin and its analogues have demonstrated issues with tolerability and failed to show efficacy [50].

**Figure 1:**
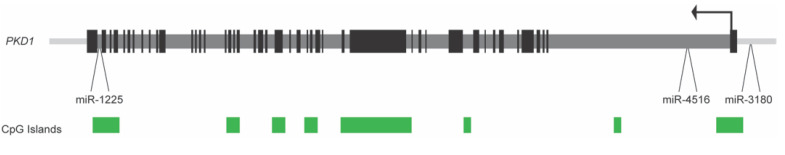
Layout of the human *PKD1* gene, showing the location of three associated miRNA genes. The *PKD1* transcription start site, and direction of transcription, is depicted by the black arrow located in exon one. Black boxes represent exons and dark grey bars are introns. Green boxes denote the location of CpG islands. miR-3180-5p is located upstream of exon one, miR-4516 is located in intron one and miR-1225 is found in intron 45. The *PKD1* gene depiction is from the UCSC Genome Browser, human genome annotation Hg19.

While miRNAs that are associated with ADPKD are located at disparate sites throughout the genome, the *PKD1* gene itself houses several miRNA genes (see Figure 1). To determine if ADPKD is associated with any changes to the expression of these intragenic loci, gene expression analysis of three miRNA found within the *PKD1* gene and its promoter (miR-1225, miR-3180-5p, and miR-4516) was performed in five human ADPKD kidney samples and three non-ADPKD samples [51]. There was little to no expression of miR-1225 and miR-3180-5p in ADPKD kidney tissue. In contrast, a wide variation in miR-4516 expression was observed in ADPKD tissues when compared to non-ADPKD kidney tissue, suggesting some dysregulation in the expression of this miRNA (which is located within intron one of *PKD1*, 2.6 kb from the transcription start site; see Figure 1). An analysis of chronic kidney disease (CKD) patient blood indicated that miR-4516 could be a marker of eGFR and renal function in patients [52], which is consistent with its variable expression level between different ADPKD patients.

### 2.3. Histone Deacetylases (HDACs)

In the nucleus, DNA is wound around histone protein octamers, which allow for the tight packaging and organisation of the DNA molecule within the nucleus. The N- and C-terminal portions of the histone proteins (histone ‘tails’), play a central role in this process, guided by a range of chemical modifications. These post-translational modifications determine how closely the histones are packaged together, in turn facilitating how freely DNA can be transcribed.

There are three main modifications to the histone tails: phosphorylation, methylation and acetylation [53]. There is little evidence of changes in histone phosphorylation and methylation in the context of ADPKD, but there are data on modification via acetylation. Acetylated histones result in relaxed chromatin (euchromatin), which is associated with greater levels of gene transcription, while deacetylation results in heterochromatin, which is tightly packed and has reduced levels of gene transcription. Histone acetylation is catalysed by histone acetyltransferases (HATs), while deacetylation requires histone deacetylases (HDACs). There are several classes of HATs and HDACs, based upon homologous sites and the target of their epigenetic effects [54].

A number of studies suggest that HDACs are regulators of PKD genes and/or the signalling pathways that are involved in cystogenesis. HDACs mediate p53-induced repression of the *PKD1* promoter, an effect which has shown to be attenuated following HDAC inhibitor treatment [55]. HDAC5 has been identified as a target of the *PKD1*-dependent fluid stress-sensing in renal epithelial cells, in which the *PKD1* protein product, polycystin, facilitates calcium influx into the cell and subsequent protein kinase C phosphorylation of HDAC5 [56]. HDAC6 is upregulated in mutant mouse *Pkd1* cells and activates a number of different factors associated with cyst growth, such as EGFR and EGF-induced β-catenin nuclear localisation. Inhibition of HDAC6 prevents the release of Ca^2+^ from the endoplasmic reticulum, consequentially attenuating cyst growth through cAMP signalling [57]. Additionally, the HDAC SIRT1 is also up-regulated in mouse *Pkd1* mutant cells. Treatment with a specific SIRT1 inhibitor decreased proliferation and increased apoptosis of cystic epithelial cells [58].

Numerous studies have identified the activity of HDACs as a driver of neoplasia, due to the aberrant expression of HDACs in tumours [59], and the ability to use post-translational modification of histones with HDACs and HATs as biomarkers in human tumour tissue [60]. As such, there are now several HDAC inhibitors that have been approved by the FDA for the use in human cancer [61]. Two of these compounds, the class I HDAC inhibitor, VPA, and class I and II inhibitor, TSA, have both been shown to reduce cyst formation and slow cyst growth in animal models of ADPKD [55,56,62,63,64]. A recent high-throughput screening platform for polycystic kidney disease drug discovery, utilising both murine and human ADPKD models, has also identified HDAC inhibitors as having differential responses between *Pkd1* mutant and wildtype cells [65].

### 2.4. DNA Methylation

DNA methylation is a stable and somatically heritable epigenetic mark which plays a regulatory role in shaping the phenotype of a cell, and has been heavily implicated in human disease [66,67]. DNA has the potential for nucleotides to be modified by the addition of a methyl group via DNA methyltransferase (DNMT) proteins. In humans, this almost exclusively occurs on cytosine residues which are immediately followed by guanine (CpGs). Mammalian DNA is methylated at 70–80% of all CpG sites in the genome [68], as DNA methylation contributes to the controlled suppression and expression of genes [36]. Clusters of CpGs are known as CpG islands, and these are often found in areas of high importance for gene expression regulation, such as at the promoter or enhancer regions of a gene.

Methylation within a gene promoter has historically been associated with repression of the gene, by reducing the ability of transcription factors to bind [69]. One mechanism of transcription factor inhibition is chromatin remodelling, suggesting that there is epigenetic cross-talk between DNA methylation and histone modification in the epigenetic control of genes [70].

Methylation of CpGs within the gene body is not as well understood as that of the promoter, but typically results in sustained or increased expression of a gene [71,72]. A variety of mechanisms have been proposed to explain this phenomenon. These all postulate that the methylated regions contain genomic elements, which are either responsible for alternative splicing, or containing transcription factors which, when hypomethylated, interfere with the host gene expression, or as residual epigenetic marks from embryonic stages of development [71].

### 2.5. DNA Methylation in Human ADPKD

The pathology of several diseases, most notably cancers, has been linked to the dysregulation of DNA methylation both globally and locally [73]. As such, inhibition of DNMTs has been developed as a therapeutic for various neoplasia [73], with the US Food and Drug Administration (FDA) having approved the DNMT inhibitor decitabine for the treatment of myelodysplastic syndromes (MDS) and chronic myelomonocytic leukemia (CMML) [74].

A 2014 paper by Woo et al. [75] was the first to report on global DNA methylation levels in ADPKD patients. This study was conducted using pyrosequencing on kidney tissue from three patients with ADPKD, compared with three samples of non-ADPKD tissue. The results from this analysis identified over 13,000 unique fragments in the genome which were differentially methylated, 91% of which were hypermethylated. Exonic regions of the genome were found to be the major targets of DNA methylation changes in ADPKD, with 5.93-fold higher methylation occurring at these regions. This group also analysed DNA methylation in the *PKD1* gene body (exon 43) and demonstrated hypermethylation in ADPKD samples, negatively correlating this with the expression of the gene. Demethylation of an immortalised cell line (WT 9–12) resulted in increased *PKD1* mRNA expression, and treatment of the cyst-forming cell line MDCK with a DNMT inhibitor repressed the growth of cysts [75].

It was theorised by Woo et al. [75] that if the hypermethylation of the ADPKD genome was resulting in cystogenesis, pharmaceuticals, such as the DNMT inhibitor decitabine, could be used for the treatment and management of ADPKD patients. A subsequent study by Woo et al. [76] on human ADPKD samples identified the *MUPCDH* (mucin-like protocadherin) gene to be hypermethylated in its promoter, and this was associated with significant repression of *MUPCDH* gene expression in ADPKD samples. The authors suggested that the methylation status of the *MUPCDH* promoter could be used as a novel epigenetic biomarker and a therapeutic target in ADPKD.

In contrast to Woo et al. [75]., Bowden et al. 2018 demonstrated a small (2%) but statistically significant hypomethylation of the genome in four ADPKD patients when compared to normal tissue (three non-ADPKD patients) [77], using Reduced Representation Bisulfite Sequencing (RRBS) as the profiling method for the genome-wide methylation analysis. Additionally, these data also revealed 13 novel regions that were differentially methylated in ADPKD tissue when compared to non-ADPKD kidney tissue. In agreement with Woo et al., the *PKD1* gene body was hypermethylated in ADPKD samples from the RRBS study, but this was associated with an increase rather than a decrease in *PKD1* mRNA expression [77]. A later study by Hajirezaei et al. [78] demonstrated *PKD1* promoter hypermethylation inversely correlated with gene expression in patient blood, further indicating a role of DNA methylation in *PKD1* expression.

DNA methylation plays a key role in gene expression modulation during disease progression, as shown in the development of aggressive metastatic tumours [79]. In the ADPKD context, cysts are believed to arise independently, and the molecular, pathological changes that underpin cyst formation are relatively poorly understood. In a recent genome-wide DNA methylation analysis of eight cysts that were derived from the same patient [80], 14.6% of the analysed fragments exhibited a substantial amount of inter-cyst DNA methylation variation, and these regions were defined as inter-cyst variants (ICVs). CpG islands (which are heavily associated with promoters and gene expression) and gene bodies demonstrated the greatest amount of variation across the ADPKD kidney. The intergenic regions were comparatively stable in their methylation levels within the cysts, suggesting that epigenetic variation (and therefore perhaps epigenomic instability) overlaps with transcriptional activity in ADPKD. Furthermore, 837 of the 5890 ICV-associated genes were also differentially methylated in ADPKD tissue, indicating that differential methylation is also a feature for at least a significant proportion of the ICVs. This work was the first study to shed light into the global methylation patterns of individual cysts and provides evidence of a role for DNA methylation changes in the development of each cyst [80].

## 3. Implications for Future DNA Methylation Research in ADPKD

To-date, relatively few studies have attempted to perform genome-wide DNA methylation analyses for ADPKD (see Table 1 for a summary). Genome-wide technologies such as RRBS, MIRA-Seq, and MeDIP-Seq have been applied to profile genomic methylation levels in ADPKD and adjacent normal samples. These methods are both comprehensive and genome-wide, however, they profile different areas of the genome based on the design of the analysis platform, as described [81]. Although these studies have been critical to provide a genomic view of DNA methylation in ADPKD, to identify several important regions, and also to reveal DNA methylation changes in *PKD1* (Table 1), these platforms are not truly a whole-genome analysis. Platforms such as Whole-Genome Bisulfite Sequencing (WGBS) are able to provide an unbiased, full DNA methylation description of ADPKD, but studies employing this technique are yet to be reported (to our knowledge).

Epigenetic processes and their associated profiling methods are complex, and utilise different approaches for deriving epigenetic marks, and each method has its own biases. The approach used therefore determines which regions of the genome are profiled [81]. Unless the method truly profiles the whole genome (such as WGBS), alternative methods could provide an overview of different regions of the genome. As each methodology used has different benefits and biases, the resulting data are not always comparable. In addition, the various methodologies also lend themselves to different statistical analyses, based on the data generated (i.e., pyrosequencing vs sanger sequencing). The statistical significance of the effects observed in these data also varies depending on the number of samples accessed. For instance, global methylation data that were generated for Woo et al. [75] and Bowden et al. [77] analysed the methylation of *PKD1* in seven and five ADPKD patients, respectively, while Hajirezaei et al. [78] generated data for 40 patients, using methylation-sensitive high-resolution melting (MS-HRM) analysis of the *PKD1* promoter [78]. This was achieved by analysing blood samples, which are more readily available. This study found hypermethylation within the promoter of *PKD1,* which was absent from previous studies. It is unclear whether blood samples have unique *PKD1* methylation patterns when compared with kidney tissue, or whether the increase in sample size led to the discovery of a smaller but significant change in DNA methylation.

A similar observation has been found in acute kidney injury (AKI) studies. A global analysis using dot blot and immunochemistry methods reported that global levels of 5-hydroxymethylcytosine were reduced in mouse kidneys affected by ischemia reperfusion injury (IRI). However, the level of DNA methylation (5-methylcytosine) was reported to be globally unchanged in ischemia reperfusion [83]. A subsequent study utilised RRBS to profile DNA methylation patterns during IRI in mouse kidneys. That study reported a large number of differentially methylated regions at different points, including substantial genome-wide DNA methylation changes in both acute and chronic IRI. The authors also demonstrated the role of promoter methylation in regulating the expression of genes associated with injury [84]. Although these results seem contradictory, it is likely that these observations are the result of utilising different genomic technologies and tissues selected for the studies.

Cell lines generated from cystic tissue have also been used to study DNA methylation in ADPKD [75]. However, the immortalisation process of deriving cell lines from tissue potentially makes them less likely to reflect the original methylome of the patient’s tissue. Data from RRBS libraries, generated from commonly used ADPKD cell lines (WT 9-7 and WT 9-12) [51], demonstrate that these cell lines do not strongly correlate (as shown by Pearson’s correlation coefficient and unsupervised hierarchical clustering) with either non-ADPKD or ADPKD renal tissue, suggesting that—either due to the immortalisation process or to long-term passaging—the source tissue methylome has not been maintained.

In contrast to that which was observed with immortalised cell lines, Kenter et al. [82] generated iPSCs from patient tissue and found concordance in methylation between the iPSCs and the source material, suggesting the iPSCs maintain an inherited epigenetic profile which could be used for future analysis. ADPKD tissue at early stages of the disease is difficult to access, and thus patient tissue is typically only available during renal transplantation following end-stage kidney disease. The tissue at this stage represents end-stage disease, and any data derived from this tissue may reflect a range of assaults to the DNA that the patient has experienced over the lifetime, rather than aberrations that are specific to the onset of disease. To address this possibility, genes associated with DMFs in ADPKD patients identified by RRBS [77] were compared with the top genes identified in the epigenomic analysis of Chronic Kidney Disease (CKD) by Chu et al. [85]. There was no overlap between the two datasets, suggesting that the RRBS DMFs identified are novel methylation differences specific to ADPKD, rather than as a consequence of CKD.

Another variable among these DNA methylation data sets is the source material. Patient tissue is typically from ESKD patients at around age 50, at which point a multitude of environmental factors are likely to have impacted each patient’s methylome (including medical interventions, such as therapeutics). Additionally, each study discussed has been generated from patient data within a defined population, which could also contribute to these differences.

ADPKD is a disease that could be characterised as cysts arising from different nephron segments. Thus, a “cyst” sample taken from tissue is very likely to represent different nephron segments and therefore could be heterogenous. Large levels of variation in DNA methylation have been observed between cysts taken from a single patient [80]. This suggests that there is the potential for a large amount of heterogeneity in tissue samples, owing to the mixture of cell composition. Therefore, ADPKD and presence of individual cyst cells provides an excellent opportunity to apply single cell epigenomic methods to unravel new biology. To our knowledge, no study has yet reported single cell epigenomic maps in ADPKD. The ability to generate genome-wide single cell maps is rapidly changing our understanding of epigenomic regulation and its implications in disease [86,87]. Single-cell DNA methylation or transcriptomic maps of individual cells from ADPKD would substantially improve our understanding of its molecular pathology. Further, detailed epigenomic profiles of early changes (that are perhaps linked with cystogenesis) and advanced changes (that are more likely to be disease progression-associated changes) in ADPKD is of critical importance, as it could improve patient management in the future.

## 4. Conclusions

Epigenetics has increasingly become an area of interest for researchers working on ADPKD, as the identification of pathways associated with cystogenesis becomes increasingly complex. New data that can shed light on additional mechanisms of disease gives us hope that additional therapeutic strategies are possible for this relatively common genetic disorder.

While novel experimental approaches have characterised the epigenome of ADPKD kidneys, more work needs to be done to establish a consensus of DNA methylation changes in ADPKD. Conflicting data on the methylation status of the genome, as well as within the *PKD1* gene itself, provides evidence of an epigenetic influence in this disease process, yet there is no clear direction for targeting treatments at this stage. However, the fact that both HDAC and DNMT inhibitors have been identified in a recent high-throughput screening platform for polycystic kidney disease drug discovery [65] suggests that targeting epigenetic changes associated with ADPKD may be a way forward for new therapeutic development.

A solution to help disentangle DNA methylation variables may be to perform multiple methodologies on biological replicates, however this does not solve the larger issue of DNA methylation methodologies each containing their own internal biases. Some standardisation of the DNA methylation field is required to develop synchrony between research groups, to more efficiently assist the analyses of patient material and to provide a clear path forward.

## Figures and Tables

**Table 1:** Summary and key findings of DNA methylation studies in ADPKD.

Study	Sample Type	Key Findings	*PKD1* Methylation	Technical Platform
Woo et al. (2014) [75]	Patient renal tissue	Gene body hypermethylation of cystogenesis-related genes, which were also downregulated in ADPKD.	Hypermethylation of the *PKD1* gene body associated with a reduction in expression.	MIRA-Seq
Woo et al. (2015) [76]	Patient renal tissue	Hypermethylation of the *MUPCDH* promoter associated with transcriptional repression. Potential novel biomarker.	Not examined	MIRA-Seq
Bowden et al. (2018) [77]	Patient renal tissue	Global hypomethylation of the ADPKD genome. Differentially methylated loci are associated with ADPKD.	Hypermethylation within *PKD1* gene body not associated with a reduction in transcription.	RRBS
Bowden et al. (2020) [80]	Patient renal tissue (individual cysts)	Methylation values in cysts reflect whole tissue RRBS data; highly variable methylation patterns in specific loci between cysts in a single patient.	Too little coverage to analyse.	RRBS
Kenter et al. (2020) [82]	iPSCs	Cells derived from patients display a methylation pattern indicative of disease-specific epigenetic memory.	No epigenetic changes to PKD-causing genes were found in iPSCs.	MeDIP-Seq
Hajirezaei et al. (2021) [78]	Patient blood	Methylation of the *PKD1* promoter inversely correlates with gene expression.	Lower *PKD1* promoter DNA methylation correlated with greater *PKD1* gene expression in patients.	MS-HRM

Abbreviations used: MIRA-Seq: Methylated-CpG Island Recovery Assay with parallel sequencing; RRBS: Reduced Representation Bisulfite Sequencing; MeDIP-Seq: Methylated DNA Immunoprecipitation Sequencing; MS-HRM: Methylation-Sensitive High Resolution Melting.
